# Overexpression of PRDX4 Modulates Tumor Microenvironment and Promotes Urethane-Induced Lung Tumorigenesis

**DOI:** 10.1155/2020/8262730

**Published:** 2020-12-28

**Authors:** Jianbo Zheng, Xin Guo, Yuka Nakamura, Xiaolei Zhou, Reimon Yamaguchi, Jing Zhang, Yasuhito Ishigaki, Hidetaka Uramoto, Sohsuke Yamada

**Affiliations:** ^1^Department of Pathology and Laboratory Medicine, Kanazawa Medical University, Ishikawa 920-0293, Japan; ^2^Department of Pediatrics, Wuhan Union Hospital, Tongji Medical College of Huazhong University of Science and Technology, Wuhan, Hubei 430022, China; ^3^Department of Pathology, Kanazawa Medical University Hospital, Ishikawa 920-0293, Japan; ^4^Department of Medical Research Institute, Kanazawa Medical University, Ishikawa 920-0293, Japan; ^5^College of Bioscience & Bioengineering, Hebei University of Science and Technology, Shijiazhuang 050018, China; ^6^Department of Dermatology, Kanazawa Medical University, Ishikawa 920-0293, Japan; ^7^Department of Thoracic Surgery, Kanazawa Medical University, Ishikawa 920-0293, Japan

## Abstract

Peroxiredoxin 4 (PRDX4), initially reported as an antioxidant, is overexpressed in lung cancer and participates in its progression. However, its role in the urethane-induced lung tumor model is undetermined. The aim of this study was to investigate the effect of PRDX4 overexpression on carcinogen-induced lung tumor development. Human PRDX4 overexpression transgenic (Tg) mice (*hPRDX4^+/+^*) and non-Tg mice were intraperitoneally injected with urethane to induce lung tumor. After 6 months, tumor formation was compared between groups and possible mechanisms for the difference in tumor development were investigated. The serum and lung PRDX4 expressions were enhanced after urethane stimulation in Tg mice. Both the average number of tumors (≥0.5 mm) and tumor diameter per mouse in the Tg group were significantly larger than in non-Tg controls, while body weight was lower in the Tg group. Compared with non-Tg controls, tumor cell proliferation was enhanced, while tumor cell apoptosis was suppressed in Tg mice. Systemic oxidative stress and oxidative stress in lung tumors were inhibited by PRDX4 overexpression. The balance of prooxidant enzymes and antioxidant enzymes was also shifted to a decreased level in Tg tumor. In lung tumor tissue, the density of microvessel penetrated into tumor was higher in the Tg group; macrophage infiltration was enhanced in Tg tumors, while there was no difference in T lymphocyte infiltration; the expressions of cytokines, including interleukin-1 beta (IL-1*β*) and matrix metallopeptidase 9 (MMP9), were elevated in Tg tumors, which resulted from enhanced phosphorylation of nuclear factor-*κ*B p65 (NF-*κ*B p65) and c-Jun, respectively. In conclusion, PRDX4 overexpression modulated tumor microenvironment and promoted tumor development in the mouse urethane-induced lung cancer model.

## 1. Introduction

Peroxiredoxin 4 (PRDX4) is ubiquitously expressed in mammalian cells [1] and is the only secretary member of the antioxidant peroxiredoxin family [2, 3]. With typical two cysteine residues, the most profound function of PRDX4 in cells is to suppress oxidative stress by eliminating H_2_O_2_. Given that oxidative stress can activate inflammation, lead to tumor transformation, and modulate tumor progression [4], the involvement of PRDX4 in tumors has been extensively studied. Increased expression of PRDX4 has been observed in many cancers, including prostate cancer [5], glioblastoma [6], oral cavity squamous cell carcinoma [7], and ovarian cancer [8], whereas decreased expression of PRDX4 in tumors was only reported in very few cancers, for example, in acute promyelocytic leukemia [9] and gastric adenocarcinoma [10]. Although many studies have shown that PRDX4 promotes tumor progression, such as the enhancement of invasion or metastasis [7, 11–15] and the augmentation of proliferation [5, 6, 15, 16], the role of PRDX4 in tumors is complicated and specific in certain tumor type. In our previous study in hepatocellular carcinoma (HCC), we found that PRDX4 inhibits the initiation of HCC but plays a complex role in tumor progression [17]. The latest study in hepatoblastoma (HB) indicated that PRDX4 promoted embryonal hepatoblastoma cell migration but induced fetal hepatoblastoma cell differentiation [18]. As different levels of reactive oxygen species (ROS), including H_2_O_2_, in tumor microenvironment exert specific effects on tumor [19], tumor microenvironment may also affect the role of PRDX4 in tumor via the crosstalk between PRDX4 and oxidative stress. In summary, PRDX4 plays different roles in different tumor contexts, including tumor histological type, tumor stage, and even tumor microenvironment, such as the oxidative stress balance.

Lung cancer is the leading cause of cancer-related deaths worldwide [20]. The majority of lung cancer patients are pathologically diagnosed with non-small-cell lung cancer (NSCLC) [21], which is associated with a poor prognosis. An early diagnosis is crucial for improving prognosis. The identification of new diagnostic markers and novel therapeutic targets can help to restrain this malignant tumor. As a protein closely related to cancer, PRDX4 has been found to be overexpressed in lung cancer, especially adenocarcinoma [22–24]. *In vitro*, knockdown of PRDX4 in the A549 lung cancer cell line results in the formation of fewer colonies and reduced Matrigel invasion [25], whereas overexpression of PRDX4 enhances anchorage-independent colony formation and Matrigel invasion [26]. *In vivo*, PRDX4-positive staining was correlated with an increased rate of recurrence and reduced disease-free survival (DFS) in squamous cell carcinoma patients [23], while our recent studies in stage I lung adenocarcinoma found that the weak expression of PRDX4 combined with a high MIB-1 labeling index predicts shortened DFS [27] and the high expression of PRDX4 combined with EGFR mutation was positively correlated with a better prognosis [28]. In comparison to massive studies on lung cancer progression, PRDX4's role in lung cancer development is still undetermined. Theoretically, PRDX4 can reduce oxidative stress and prevent lung carcinogenesis, but PRDX4 can be secreted into extracellular space and extracellular PRDXs have been demonstrated to be capable of activating the expression of inflammatory cytokines and initiating postischemic inflammation in the brain [29]. Secreted or extracellular PRDX4 may activate inflammation, which can lead to oncogenesis, like its family member PRDX1 [30]. It has been proven that PRDX6, another PRDX family member, promotes the development of urethane-induced lung adenocarcinoma in mice [31].

As a carcinogen, urethane has been widely used to induce pulmonary adenoma in mice, which mimics human lung adenocarcinoma and offers important insights in tumor development [32]. In the present study, we examined the effect of PRDX4 overexpression on the development of lung adenoma induced by urethane by comparing tumor formation between PRDX4 transgenic (Tg) mice and non-Tg mice.

## 2. Materials and Methods

### 2.1. Ethics Statement

All animal experiments were approved by the Ethics Committee of Animal Care and Experimentation, Kanazawa Medical University, Japan, and were carried out according to institutional guidelines for animal experiments and the law (no. 105) and notification (no. 6) of the Japanese government. Isoflurane was used as the euthanasia agent in animal experiments.

### 2.2. Animal Experiments

The construction of hPRDX4 Tg mice on C57BL/6 background was detailed in our previous study [33]. C57BL/6 mice were purchased from Charles River Laboratories (Yokohama, Japan) as non-Tg control. All mice were housed and bred under specific-pathogen-free conditions at the Animal Research Center of Kanazawa Medical University. All mice were kept in a room with relatively constant temperature (21-23°C), humidity (50-60%), and 12-hour light/dark circle. They received a standard chow diet and purified tap water *ad libitum*. PRDX4 Tg and non-Tg male mice (8 weeks old, *n* = 15 for each group) were subjected to intraperitoneal injection of urethane (1 g/kg in 100 *μ*l saline) once per week for 16 consecutive weeks. Body weights were recorded weekly. Mice were sacrificed at 6 months after the initial injection. Lungs were harvested, and the number of tumors and their diameter on the lung surface were measured. Serum was also collected after centrifugation. Another group of mice (8 weeks old, *n* = 3 for each group) received one urethane injection every other day and were sacrificed after one week (short-term urethane stimulation trial).

### 2.3. Histology and Immunohistochemistry

Harvested lungs were immersed in 4% paraformaldehyde for over 24 hours and then embedded in paraffin. Sections (thickness: 3-5 *μ*m) were subjected to hematoxylin and eosin (H&E) and immunohistochemical (IHC) staining. For IHC staining, the procedure was as follows: (1) deparaffinization and rehydration; (2) 0.5% hydrogen peroxide blocking for 15 minutes at room temperature; (3) antigen retrieval: heat-mediated antigen retrieval (trypsin-mediated antigen retrieval was exclusively applied for PRDX4); (4) 3% bovine serum albumin blocking for 30 minutes at room temperature; (5) primary antibody incubation overnight at 4°C; (6) secondary antibody (Histofine Simple Stain MAX-PO424152) staining for 30 minutes at room temperature; and (7) 3,3′-diaminobenzidine (DAB) imaging and hematoxylin counterstaining. H&E and IHC staining images were captured and quantitatively analyzed using the NanoZoomer Digital Pathology Virtual Slide Viewer software program (Hamamatsu Photonics Corp., Hamamatsu, Japan).

### 2.4. Terminal Deoxynucleotidyl Transferase dUTP Nick-End Labeling (TUNEL) Assay

The In Situ Cell Death Detection Kit, POD (Roche, 11684817910), was employed for this assay. Formalin-fixed and paraffin-embedded sections (thickness: 3-5 *μ*m) were stained according to the manufacturer's instructions. After DAB imaging and hematoxylin counterstaining, the final staining images were captured and quantitatively analyzed using the NanoZoomer Digital Pathology Virtual Slide Viewer software program (Hamamatsu Photonics Corp., Hamamatsu, Japan).

### 2.5. PRDX4 (Human) ELISA Assay

Blood was drained from the mouse axillary vein and then centrifuged. Serum was obtained and diluted 20-folds for the detection of hPRDX4. A PRDX4 (Human) ELISA kit (Abnova, KA2121) was used for this analysis. The absorbance value on 450 nm was detected, and final serum hPRDX4 concentration was calculated.

### 2.6. Mouse Whole-Transcript Array

GeneChip® Mouse Gene 2.0 ST Array (Affymetrix, Inc.) was used for mouse transcript analysis. Tumors were first resected from lung tissue, and then, RNA was extracted. After the examination of RNA quality, the whole-transcript array analysis was performed in a step-by-step manner according to the manufacturer's instructions. Three mice per each group were included in this analysis. Data were analyzed by Ingenuity® pathway analysis.

### 2.7. Real-Time Reverse Transcription Polymerase Chain Reaction (RT-PCR)

Total RNA was extracted from lung tumor tissue using the ReliaPrep™ RNA TissueMiniprep System (Promega Corporation, USA) and was stored at -80°C. Conversion to cDNA was conducted by a High Capacity RNA-to-cDNA Kit (Life Technologies). cDNA was amplified (40 cycles) in the Applied Biosystems™ QuantStudio™ 12K Flex Real-Time PCR System with the help of TaqMan gene expression assays (Life Technologies). Each sample was tested in triplicate for housekeeping and target genes. The Eukaryotic 18S ribosomal RNA (18S rRNA) Endogenous Control (Applied Biosystems®, 4333760T) was used as an endogenous control. The 2^-*ΔΔCT*^method was used for relative quantification in data analysis. Custom-produced primers and TaqMan probes for target gene amplification were purchased from Life Technologies. Target genes are as follows: *B-cell lymphoma 2* (*BCL2*), *BCL2-like 1* (*BCL2L1*), *BCL2-associated X* (*BAX*), *BCL2 homologous antagonist/killer* (*BAK*), *BCL2 associated agonist of cell death* (*BAD*), *IL-1β*, *IL-6*, *tumor necrosis factor-α* (*TNF-α*), *interferon gamma* (*IFN-γ*), *NF-κB*, *nuclear factor erythroid 2-related factor 2* (*Nrf2*), *inducible nitric oxide synthase* (*iNOS*), *IL-1R1*, *MMP3*, *MMP9*, *MMP12*, *MMP13*, *catalase* (*CAT*), *superoxide dismutase* (*SOD*) *1*, *SOD2*, and *SOD3.* The pairs of specific primers and probes for all of the genes were described in our previous papers [33, 34].

### 2.8. Western Blotting (WB)

Lung tumor tissue was homogenized in RIPA Lysis and Extraction Buffer (Thermo Fisher Scientific) to which protease inhibitor had been added. 10-50 *μ*g protein extract was loaded onto 5-12.5% SDS-PAGE gels (Bio-Rad) and transferred to nitrocellulose membranes (Bio-Rad) after electrophoresis. Membranes were blocked in 3% bovine serum albumin (BSA) and then probed with corresponding antibodies. The ImageJ software program was used for band quantification.

### 2.9. Thiobarbituric Acid Reactive Substance (TBARS) Assay

The TBARS Assay Kit (Cayman Chemical, USA) was used for the quantification of malondialdehyde (MDA) in mouse serum. The detailed protocol is listed in the product datasheet.

### 2.10. Antibodies Used in IHC and WB

PRDX4 (PA3-753, Thermo Fisher, 1 : 1000 in IHC and WB), proliferating nuclear antigen (PCNA) (sc56, Santa Cruz Biotechnology, 1 : 200 in IHC and 1 : 1000 in WB), 8-OHdG (N45.1, Japan Institute for the Control of Aging; 1 : 200 in IHC), Mac-2 (CL8942LE, Cedarlane Laboratories, 1 : 1000 in IHC), CD3 (ab5690, Abcam, 1 : 100 in IHC), CD31 (DIA-310, Dianova, 1 : 20 in IHC), cleaved caspase-3 (# 9661S, Cell Signaling Technology, 1 : 200 in IHC and 1 : 1000 in WB), IL-1*β* (#12242S, Cell Signaling Technology, 1 : 1000 in WB), NF-*κ*Bp65 (# 8242S, Cell Signaling Technology, 1 : 1000 in WB), phospho-NF-*κ*B p65 (#3033S, Cell Signaling Technology, 1 : 1000 in WB), TNF-*α* (#3707S, Cell Signaling Technology, 1 : 1000 in WB), phospho-c-Jun (Ser73) (#3270, Cell Signaling Technology, 1 : 1000 in WB), c-Jun (#9165, Cell Signaling Technology, 1 : 1000 in WB), MMP3 (ab52915, 1 : 1000 in WB), MMP9 (ab38898, Abcam, 1 : 1000 in WB), nicotinamide adenine dinucleotide phosphate oxidase (NOX) 2 (ab129068, Abcam, 1 : 5000 in WB), NOX4 (ab133303, Abcam, 1 : 2000 in WB), CAT (ab16731, Abcam, 1 : 2000 in WB), SOD1 (ab13498, Abcam, 1 : 2000 in WB), SOD2 (ab13533, Abcam, 1 : 5000 in WB), and SOD3 (ab83108, Abcam, 1 : 1000 in WB) were used.

### 2.11. Statistical Analysis

Variables were expressed as the mean ± SD. Two-tailed independent Student's *t*-test was employed for comparisons. All statistical analyses were performed using the SPSS statistical software package, version 16.0. Two-sided *p* values of <0.05 were considered to indicate statistical significance.

## 3. Results

### 3.1. Elevated Expression of PRDX4 in Tg Mice after Urethane Stimulation

**Figure 1:**
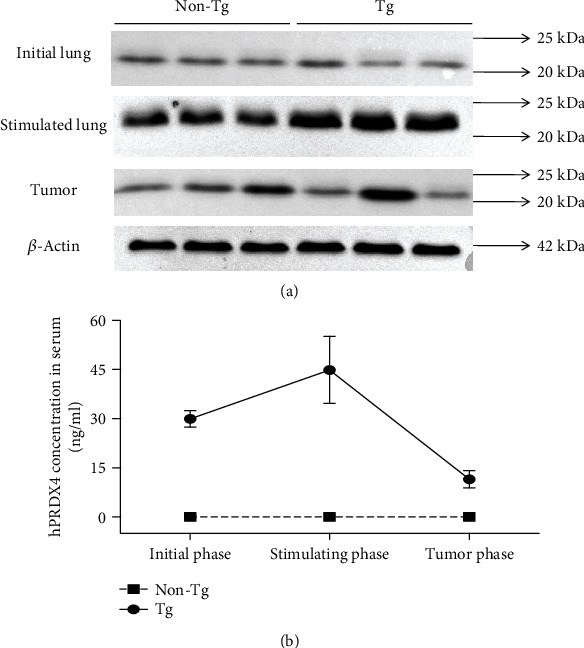
Elevated expressions of PRDX4 in Tg mice after urethane stimulation. (a) Comparisons of PRDX4 expression between Tg and non-Tg controls in initial lung tissues, in lung tissues after urethane stimulation, and in lung tumor tissues, respectively, by Western blotting. (b) Serum hPRDX4 expressions detected by the ELISA method in the process of tumor formation. The values represent mean ± SD, and the experiments shown were repeated in triplicate with similar results.

Before urethane injection, no significant PRDX4 expression difference was found in the lungs between Tg mice and non-Tg mice. We also found no difference in the expression of PRDX4 between lung tumors from these two groups. However, lung PRDX4 expression was significantly higher in Tg mice than in non-Tg controls after short-term urethane stimulation (one injection every other day for one week) (Figure 1(a)). An intermediate level of serum hPRDX4 was found in Tg mice before urethane injection (29.93 ± 2.50 ng/ml). The level was increased after short-term urethane stimulation (44.82 ± 10.22 ng/ml) and decreased after tumor formation (11.51 ± 2.62 ng/ml), which may be explained by senescence and cancer-associated cachexia. No or very weak serum hPRDX4 was detected in non-Tg mice (Figure 1(b)).

### 3.2. Promotion of Tumor Development in PRDX4 Tg Mice

**Figure 2:**
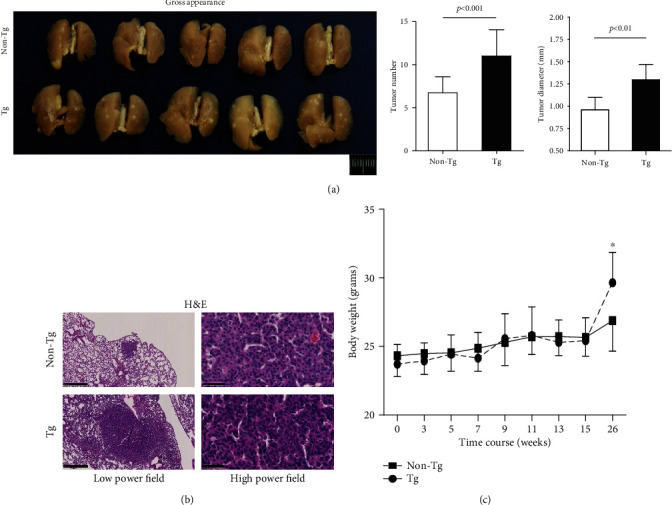
Promotion of lung tumor development in Tg mice. (a) A gross view of lung tumors (bar = 1 cm). Surface tumor number and diameter were measured at the time of sacrifice; the results are expressed in mean ± SD. (b) A microscopic view of lung tumors in hematoxylin and eosin (H&E) staining (low power field, bar = 500 *μ*m; high power field, bar = 50 *μ*m). (c) The changes of mouse body weight in tumor development. Mouse body weight was recorded during the urethane injection phase and at the time of sacrifice. Data are shown as mean ± SD. The independent samples *t*-test was used for analysis. ^∗^*p* < 0.05.

The number of tumors (≥0.5 mm) and their diameter on the lung surface were measured after mice were sacrificed. The average number of tumors per mouse in PRDX4 Tg mice was significantly greater in comparison to non-Tg controls (Tg 11.00 ± 3.06 vs. non-Tg 6.73 ± 1.85, *p* < 0.001). Moreover, the average tumor diameter (mm) in the Tg group was also larger than in non-Tg controls (Tg 1.30 ± 0.17 vs. non-Tg 0.96 ± 0.14, *p* < 0.01) (Figure 2(a)). H&E staining further confirmed this finding, with increased tumor formation in Tg mice (Figure 2(b)). In accordance with increased tumor formation, the average body weight (g) of Tg mice was significantly smaller than in non-Tg controls at the time of sacrifice (Tg 26.88 ± 2.23 vs. non-Tg 29.64 ± 2.22, *p* < 0.05) (Figure 2(c)).

### 3.3. Suppressed Tumor Apoptosis and Enhanced Tumor Proliferation in Tg Mice

**Figure 3:**
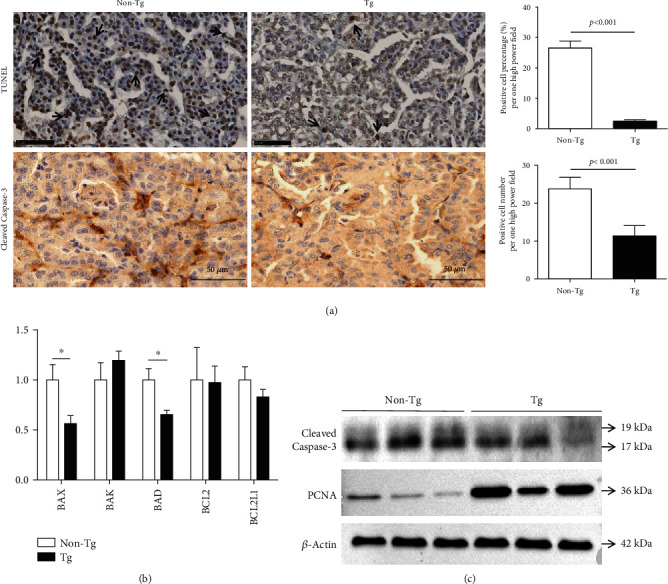
Suppression of tumor cell apoptosis and enhancement of tumor proliferation in Tg tumors. (a) Representative microscopic views of TUNEL assay and cleaved caspase-3 immunohistochemical staining (bar = 50 *μ*m). Formalin-fixed paraffin-embedded sections (thickness: 3-5 *μ*m) were used for staining. Data are shown as mean ± SD; the independent samples *t*-test was used for analysis. (b) Analysis of apoptosis-related gene expressions by RT-PCR. The 2^-*ΔΔCT*^ method was used for relative quantification of genes. Data are shown as mean ± SD; the independent samples *t*-test was used for analysis. ^∗^*p* < 0.05. (c) Protein expressions of cleaved caspase-3 and proliferating cell nuclear antigen (PCNA) by Western blotting.

The TUNEL assay showed that tumor cell apoptosis was significantly suppressed in Tg mice when compared with non-Tg controls. Immunohistochemical staining of cleaved caspase-3 also revealed that fewer cells underwent apoptosis in Tg tumors (Tg 11.40 ± 2.70 vs. non-Tg 23.80 ± 3.03, *p* < 0.001). Analysis of apoptosis-related genes by RT-PCR showed proapoptotic genes (*BAX*, *BAD*) were decreased in Tg tumor while no significant difference was found in antiapoptotic genes (*BCL2*, *BCL2L1*), leading to an imbalance which was in favor of antiapoptosis in Tg tumors. Western blotting showed that the expression of cleaved caspase-3 was significantly weaker in Tg tumors than in non-Tg control tumors, while the proliferating cell nuclear antigen (PCNA) expression in Tg tumors was stronger than non-Tg control tumors (Figure 3), indicating tumor apoptosis was suppressed and tumor proliferation was enhanced in Tg mice.

### 3.4. Increased Microvascular Permeability and Macrophage Infiltration in Lung Tumor of PRDX4 Tg Mice

**Figure 4:**
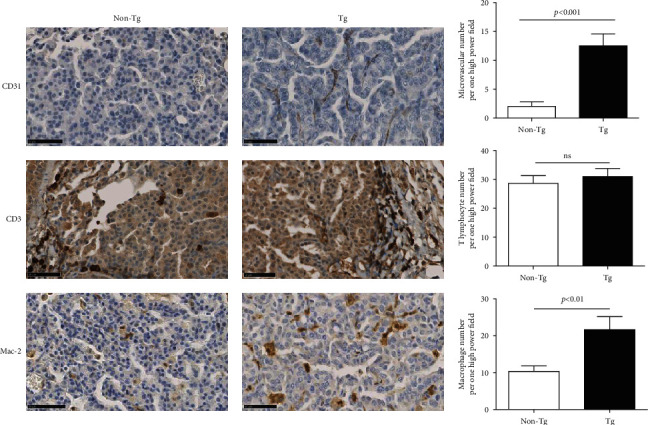
Increased microvascular permeability and macrophage infiltration in Tg tumors. Microvascular permeability was represented by microvascular number per one high power field. Infiltration of T lymphocyte and macrophage was represented by CD3-positive cell and Mac-2-positive cell presented in tumor, respectively. Bar = 50 *μ*m. Data are shown as mean ± SD. The independent samples *t*-test was used for analysis.

In IHC staining, the number of microvessels per one high power field, which were shaped by CD31-positive cells, in the lung tumors of Tg mice was significantly greater than non-Tg mice (Tg 12.50 ± 2.08 vs. non-Tg 2.00 ± 0.82, *p* < 0.001), indicating increased microvascular permeability. CD3 lymphocytes were mainly found in the marginal area of tumor, and there was no significant difference between two groups. Macrophages (Mac-2-positive cells) infiltrated into tumor and were more frequently presented in Tg tumors than in non-Tg controls (Tg 21.67 ± 3.51 vs. non-Tg 10.33 ± 1.53, *p* < 0.01) (Figure 4).

### 3.5. Elevated MMP9 and IL-1*β* Expressions in Tg Tumor

**Figure 5:**
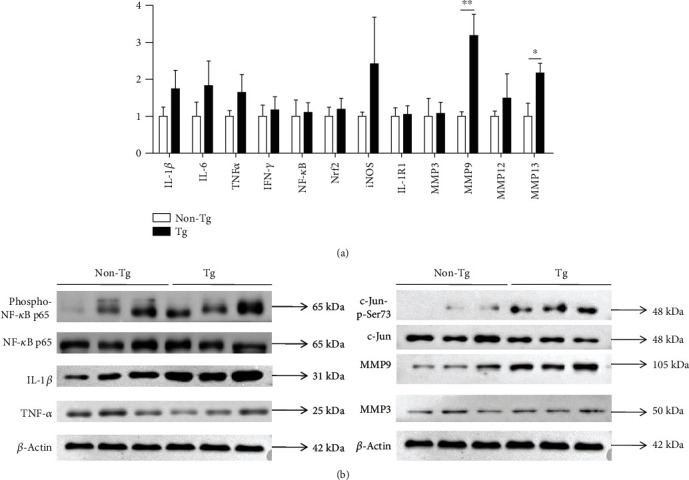
Elevated expressions of matrix metallopeptidase 9 (MMP9) and interleukin-1*β* (IL-1*β*) in Tg tumors. (a) Gene expression analysis of cytokines and MMP by RT-PCR in tumor tissues. The 2^-*ΔΔ*CT^ method was used for relative quantification of genes. Data are shown as mean ± SD. The independent samples *t*-test was used for analysis. ^∗^*p* < 0.05 and ^∗∗^*p* < 0.01. (b) Protein expression analysis of IL-1*β*, MMP9, and some other proteins by Western blotting.

Mouse whole-transcript array revealed that there were significant differences in 17 transcripts between groups when a 1.5-fold change was used as the threshold. Only 4 transcripts (*Hspa1b|Hspa1a*, *Igkv12-44*, *Igkv3-5*, and *Igkv4-91*) showed 2-fold change (Supplementary Table 1). RT-PCR revealed a >3-fold increase in the expression of *MMP9* and an over 2-fold increase in the expression of *MMP13* in Tg tumors in comparison to non-Tg controls. Cytokine expression analysis, including *IL-1β*, *IL-6*, *TNF-α*, and *iNOS*, also showed a tendency toward increased expression in Tg tumors, though no statistically significant difference was reached (Figure 5(a)). WB showed the obvious elevation of MMP9 and IL-1*β* expressions in Tg mice, whereas no difference was found in TNF-*α* or MMP3. The phosphorylation of NF-*κ*B p65 and c-Jun was significantly enhanced in Tg tumor tissue, indicating activation of NF-*κ*B and activator protein 1 (AP-1) pathway (Figure 5(b)).

### 3.6. Inhibition of Oxidative Stress in Tg Mice

**Figure 6:**
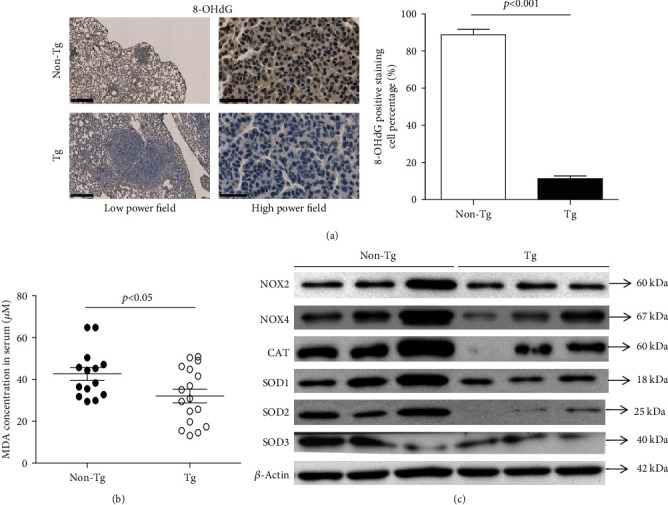
Inhibition of oxidative stress in Tg mice. (a) Representative views of 8-OHdG immunohistochemical staining (low power field, bar = 500 *μ*m; high power field, bar = 50 *μ*m). (b) Serum malondialdehyde (MDA) level in Tg mice and non-Tg controls at the time of sacrifice. (c) Protein expressions of prooxidant enzymes and antioxidant enzymes by Western blotting. NOX: nicotinamide adenine dinucleotide phosphate oxidase; CAT: catalase; SOD: superoxide dismutase. Data are shown as mean ± SD. The independent samples *t*-test was used for analysis.

IHC staining of 8-OHdG in tumors revealed that there were fewer positively stained cells in Tg tumors in comparison to non-Tg tumors (positively stained cell proportion: Tg 11.25% ± 1.50% vs. non-Tg 88.75% ± 3.00%, *p* < 0.001), indicating obvious inhibition of local oxidative stress in Tg tumors (Figure 6(a)). TBARS assay showed that the MDA concentration (*μ*M) in Tg serum was significantly lower than in non-Tg controls after tumor formation (Tg 32.10 ± 13.39 vs. non-Tg 42.60 ± 11.58, *p* < 0.05), indicating a decrease in systemic oxidative stress (Figure 6(b)). RT-PCR showed gene expressions of antioxidant enzymes (*SOD1* and *SOD2*) were decreased in Tg tumors when compared with non-Tg controls (Supplementary Figure 1). Expression analysis of prooxidant enzymes (NOX2 and NOX4) and antioxidant enzymes (CAT, SOD1, SOD2, and SOD3) by Western blotting revealed both enzyme expressions were lower in Tg tumors than non-Tg controls, indicating a decreased balance point in the Tg group (Figure 6(c)).

## 4. Discussion

In the present study, we demonstrated the overexpression of PRDX4, the only secreted member of the PRDX family, promoting tumor development in urethane-induced lung adenoma. In our study, greater numbers of lung carcinoma tumors and larger tumors were found in PRDX4 Tg mice, accompanied by enhanced macrophage infiltration and the elevated expression of IL-1*β* and MMP9 in tumor microenvironment. Tumor microenvironment is the surrounding environment where tumor develops and survives. Its components include immune cells, cytokines, and even products of oxidative stress. Each component is critical for tumorigenesis. It is widely accepted that macrophages participate in tumor initiation and progression [35–39]. In the phase of oncogenesis, inflammatory cells including macrophages contribute to genetic mutations and instability [39]. In the tumor progression phase, tumor-associated macrophages promote angiogenesis and remodel extracellular matrix [40–43]. Previous studies on the role of macrophages in the urethane-induced mouse lung adenoma model verified that macrophages promote both cancer carcinogenesis [44] and progression [45]. In our present study, more macrophages emerged in tumor tissue, which may partly explain the effect of PRDX4 overexpression in the promotion of tumor development. Although no key players in lung cancer development were found to be significantly changed after PRDX4 overexpression in the whole-transcript array, other analyses (RT-PCR and WB) revealed some important positive findings. As a critical cytokine in inflammation, IL-1*β* has been demonstrated to efficiently promote chemical-induced carcinogenesis [46, 47]. In this study, the expression of IL-1*β* was obviously increased in PRDX4 Tg tumor microenvironment. The elevation of IL-1*β* may also account for enhanced lung tumor development in Tg mice. The relationship between PRDX4 overexpression and AP-1 activation has been well elucidated in the A549 lung cancer cell line [25, 26]. PRDX4 was critical for the activation of AP-1 signaling, and depletion of PRDX4 leads to reduced phosphorylation of c-Jun and decreased expression of MMP9, which contributes to the malignancy of human lung cancer cells. The present study verified a similar relationship in an animal model of carcinogen-induced lung cancer. With the overexpression of PRDX4 in mice, increased phosphorylation of c-Jun (Ser 73) was observed, and the expression of MM9 was elevated in sequence. MMP9 was critical for tumor angiogenesis and extracellular matrix remodeling. In accordance with the elevated MMP9 expression, the expression of CD31 was observed to be increased in PRDX4 Tg tumor tissue, indicating a higher density of microvessels which benefits the development of lung adenoma in mice. All of the above findings indicated that a changing microenvironment, which favored tumor development, was created after the overexpression of PRDX4. One seemingly contradictory phenomenon in our study is that the 8-OHdG and MDA levels were decreased in Tg mice, which represents a lower oxidative stress level and theoretically leads to the prevention of tumor initiation. However, the role of oxidative stress in cancer development is complicated and elevated oxidative stress potentially plays a uniquely dual (double-faced) role in the different (early to late) stages of lung tumorigenesis, including protumorigenic and tumor-suppressing effects. It is still very difficult to accurately define which level of oxidative stress is mild, moderate, or severe in these *in vivo* experiments and which level is the most beneficial in a specified tumor development. In our study, the relatively lower oxidative stress level in Tg mice may favor lung tumor development via the promotion of tumor proliferation and the inhibition of tumor apoptosis. Another oxidative stress-related finding in our study is that prooxidant enzymes (NOX2 and NOX4) and antioxidant enzymes (CAT and SOD1-3) were both decreased in Tg tumors. These changes may be because of the inhibition of oxidative stress after PRDX4 overexpression. This shift in prooxidant enzyme and antioxidant enzyme balance may cause the imbalance of proapoptotic (*BAX* and *BAD*) and antiapoptotic genes (*BCL2* and *BCL2L1*), resulting in a tumor microenvironment which is in favor of surviving and resistant to apoptosis in Tg tumor.

The balance of oxidative stress and antioxidants is crucial in maintaining our body health. Imbalance can lead to pathological conditions, including inflammation and cancer [4]. Although antioxidant therapy has been demonstrated to be beneficial in inflammation and ischemia/reperfusion injury [48], certain antioxidants may exert a totally reverse effect. A study on stroke revealed that PRDXs were key initiators of postischemic inflammation [29]. Another study found that PRDXs released by cancer cells can mediate osteoclastogenesis [14], a process mainly performed by macrophages. Our recent study demonstrated that PRDX4 overexpression was associated with the aggravation of inflammation in idiopathic pulmonary fibrosis [49]. Briefly, antioxidant therapy should be approached with caution, even in inflammatory disease, and the treatment should be based on the administration of certain antioxidants and for specified inflammation. Given the conflicting effect of antioxidant therapy in inflammation and the close relationship between tumor and inflammation, it is easy to understand the diverse roles of antioxidants in tumor development. Antioxidants like PRDX4 may suppress liver inflammation and prevent hepatocellular carcinoma but may exacerbate pulmonary inflammation and promote the development of lung adenocarcinoma. Even in a certain type of tumor, antioxidant supplementation may have totally different effects according to different tumor contexts. From our series of studies about the role of PRDX4 in lung cancer, PRDX4 supplementation may be beneficial in some patients who are in the early stage of lung adenocarcinoma, but it may promote cancer progression in other patients and should be avoided in cancer prevention because of different tumor contexts.

## 5. Conclusions

PRDX4 overexpression promotes urethane-induced lung tumorigenesis, and the alteration of microenvironment caused by a high expression of this antioxidant enzyme may play important roles in this process. The results of the present study could provide novel insights in relation to antioxidant therapy for lung cancer.

## Data Availability

The primary data used to support the findings of this study are available from the corresponding author upon request.

## Supplementary Materials

Supplementary Materials Supplementary Table 1: differential expression transcripts in the whole-transcript array (fold change > 1.5). Supplementary Figure 1: analysis of antioxidant enzyme gene expressions by RT-PCR in tumor tissues; *CAT*: catalase, *SOD*: superoxide dismutase. The 2^-*ΔΔ*CT^ method was used for relative quantification of genes. Data are shown in mean ± SD. The independent samples *t*-test was used for analysis. ^∗^*p* < 0.05.

## References

[1] Rhee S. G., Chae H. Z., Kim K. (2005). Peroxiredoxins: a historical overview and speculative preview of novel mechanisms and emerging concepts in cell signaling. *Free Radical Biology and Medicine*.

[2] Fujii J., Ikeda Y., Kurahashi T., Homma T. (2015). Physiological and pathological views of peroxiredoxin 4. *Free Radical Biology and Medicine*.

[3] Yamada S., Guo X. (2018). Peroxiredoxin 4 (PRDX4): its critical in vivo roles in animal models of metabolic syndrome ranging from atherosclerosis to nonalcoholic fatty liver disease. *Pathology International*.

[4] Reuter S., Gupta S. C., Chaturvedi M. M., Aggarwal B. B. (2010). Oxidative stress, inflammation, and cancer: how are they linked?. *Free Radical Biology and Medicine*.

[5] Ummanni R., Barreto F., Venz S. (2012). Peroxiredoxins 3 and 4 are overexpressed in prostate cancer tissue and affect the proliferation of prostate cancer cells in vitro. *Journal of Proteome Research*.

[6] Kim T. H., Song J., Alcantara Llaguno S. R. (2012). Suppression of peroxiredoxin 4 in glioblastoma cells increases apoptosis and reduces tumor growth. *PLos One*.

[7] Chang K.-P., Yu J.-S., Chien K.-Y. (2011). Identification of PRDX4 and P4HA2 as metastasis-associated proteins in oral cavity squamous cell carcinoma by comparative tissue proteomics of microdissected specimens using iTRAQ technology. *Journal of Proteome Research*.

[8] Pylvas M., Puistola U., Kauppila S., Soini Y., Karihtala P. (2010). Oxidative stress-induced antioxidant enzyme expression is an early phenomenon in ovarian carcinogenesis. *European Journal of Cancer*.

[9] Palande K. K., Beekman R., van der Meeren L. E., Beverloo H. B., Valk P. J. M., Touw I. P. (2011). The antioxidant protein peroxiredoxin 4 is epigenetically down regulated in acute promyelocytic leukemia. *PLos One*.

[10] Jang J. S., Cho H. Y., Lee Y. J., Ha W. S., Kim H. W. (2004). The differential proteome profile of stomach cancer: identification of the biomarker candidates. *Oncology Research*.

[11] Basu A., Banerjee H., Rojas H. (2011). Differential expression of peroxiredoxins in prostate cancer: consistent upregulation of PRDX3 and PRDX4. *The Prostate*.

[12] Song P., Bao H., Yu Y. (2009). Comprehensive profiling of metastasis-related proteins in paired hepatocellular carcinoma cells with different metastasis potentials. *Proteomics Clinical Applications*.

[13] Li M., Lin Y.-M., Hasegawa S. (2004). Genes associated with liver metastasis of colon cancer, identified by genome-wide cDNA microarray. *International Journal of Oncology*.

[14] Rafiei S., Tiedemann K., Tabaries S., Siegel P. M., Komarova S. V. (2015). Peroxiredoxin 4: a novel secreted mediator of cancer induced osteoclastogenesis. *Cancer Letters*.

[15] Wang W., Shen X.-B., Huang D.-B., Jia W., Liu W. B., He Y. F. (2019). Peroxiredoxin 4 suppresses anoikis and augments growth and metastasis of hepatocellular carcinoma cells through the *β*-catenin/ID2 pathway. *Cellular Oncology*.

[16] Ummanni R., Mundt F., Pospisil H. (2011). Identification of clinically relevant protein targets in prostate cancer with 2D-DIGE coupled mass spectrometry and systems biology network platform. *PLoS One*.

[17] Guo X., Noguchi H., Ishii N. (2019). The association of peroxiredoxin 4 with the initiation and progression of hepatocellular carcinoma. *Antioxidants & Redox Signaling*.

[18] Zheng J.-B., Guo X., Shioya A. (2020). Peroxiredoxin 4 promotes embryonal hepatoblastoma cell migration but induces fetal cell differentiation. *American Journal of Translational Research*.

[19] Jia W., Chen P., Cheng Y. (2019). PRDX4 and its roles in various cancers. *Technology in Cancer Research & Treatment*.

[20] Global Burden of Disease Cancer Collaboration (2017). Global, regional, and national cancer incidence, mortality, years of life lost, years lived with disability, and disability-adjusted life-years for 32 cancer groups, 1990 to 2015: a systematic analysis for the global burden of disease study. *JAMA Oncology*.

[21] Vincent R. G., Pickren J. W., Lane W. W. (1977). The changing histopathology of lung cancer: a review of 1682 cases. *Cancer*.

[22] Pastor M. D., Nogal A., Molina-Pinelo S., Carnero A., Paz-Ares L. (2013). Proteomic biomarkers in lung cancer. *Clinical and Translational Oncology*.

[23] Hwang J. A., Song J. S., Yu D. Y. (2015). Peroxiredoxin 4 as an independent prognostic marker for survival in patients with early-stage lung squamous cell carcinoma. *International Journal of Clinical and Experimental Pathology*.

[24] Lehtonen S. T., Svensk A.-M., Soini Y. (2004). Peroxiredoxins, a novel protein family in lung cancer. *International Journal of Cancer*.

[25] Wei Q., Jiang H., Xiao Z. (2011). Sulfiredoxin-peroxiredoxin IV axis promotes human lung cancer progression through modulation of specific phosphokinase signaling. *Proceedings of the National Academy of Sciences of the United States of America*.

[26] Jiang H., Wu L., Mishra M., Chawsheen H. A., Wei Q. (2014). Expression of peroxiredoxin 1 and 4 promotes human lung cancer malignancy. *American Journal of Cancer Research*.

[27] Shioya A., Guo X., Motono N. (2018). The combination of weak expression of PRDX4 and very high MIB-1 labelling index independently predicts shorter disease-free survival in stage I lung adenocarcinoma. *International Journal of Medical Sciences*.

[28] Mizutani K., Guo X., Shioya A. (2019). The impact of PRDX4 and the EGFR mutation status on cellular proliferation in lung adenocarcinoma. *International Journal of Medical Sciences*.

[29] Shichita T., Hasegawa E., Kimura A. (2012). Peroxiredoxin family proteins are key initiators of post-ischemic inflammation in the brain. *Natural Medicines*.

[30] Ishii T., Warabi E., Yanagawa T. (2012). Novel roles of peroxiredoxins in inflammation, cancer and innate immunity. *Journal of Clinical Biochemistry and Nutrition*.

[31] Yun H. M., Park K. R., Park M. H. (2015). PRDX6 promotes tumor development via the JAK2/STAT3 pathway in a urethane-induced lung tumor model. *Free Radical Biology & Medicine*.

[32] Stoner G. D. (1998). Introduction to mouse lung tumorigenesis. *Experimental Lung Research*.

[33] Ding Y., Yamada S., Wang K.-Y. (2010). Overexpression of peroxiredoxin 4 protects against high-dose streptozotocin-induced diabetes by suppressing oxidative stress and cytokines in transgenic mice. *Antioxidants & Redox Signaling*.

[34] Nawata A., Noguchi H., Mazaki Y. (2016). Overexpression of peroxiredoxin 4 affects intestinal function in a dietary mouse model of nonalcoholic fatty liver disease. *PLoS One*.

[35] Mantovani A., Allavena P., Sica A., Balkwill F. (2008). Cancer-related inflammation. *Nature*.

[36] Sica A., Allavena P., Mantovani A. (2008). Cancer related inflammation: the macrophage connection. *Cancer Letters*.

[37] Pollard J. W. (2009). Trophic macrophages in development and disease. *Nature Reviews Immunology*.

[38] Karin M., Lawrence T., Nizet V. (2006). Innate immunity gone awry: linking microbial infections to chronic inflammation and cancer. *Cell*.

[39] Biswas S. K., Mantovani A. (2010). Macrophage plasticity and interaction with lymphocyte subsets: cancer as a paradigm. *Nature Immunology*.

[40] Clear A. J., Lee A. M., Calaminici M. (2010). Increased angiogenic sprouting in poor prognosis FL is associated with elevated numbers of CD163+ macrophages within the immediate sprouting microenvironment. *Blood*.

[41] Murdoch C., Muthana M., Coffelt S. B., Lewis C. E. (2008). The role of myeloid cells in the promotion of tumour angiogenesis. *Nature Reviews Cancer*.

[42] Lin E. Y., Li J. F., Gnatovskiy L. (2006). Macrophages regulate the angiogenic switch in a mouse model of breast cancer. *Cancer Research*.

[43] Lin E. Y., Nguyen A. V., Russell R. G., Pollard J. W. (2001). Colony-stimulating factor 1 promotes progression of mammary tumors to malignancy. *The Journal of Experimental Medicine*.

[44] Zaynagetdinov R., Sherrill T. P., Polosukhin V. V. (2011). A critical role for macrophages in promotion of urethane-induced lung carcinogenesis. *Journal of immunology*.

[45] Fritz J. M., Tennis M. A., Orlicky D. J. (2014). Depletion of tumor-associated macrophages slows the growth of chemically induced mouse lung adenocarcinomas. *Frontiers in Immunology*.

[46] Apte R. N., Dotan S., Elkabets M. (2006). The involvement of IL-1 in tumorigenesis, tumor invasiveness, metastasis and tumor-host interactions. *Cancer Metastasis Reviews*.

[47] Narayan C., Kumar A. (2012). Constitutive over expression of IL-1*β*, IL-6, NF-*κ*B, and Stat3 is a potential cause of lung tumorgenesis in urethane (ethyl carbamate) induced Balb/c mice. *Journal of Carcinogenesis*.

[48] Cuzzocrea S., Riley D. P., Caputi A. P., Salvemini D. (2001). Antioxidant therapy: a new pharmacological approach in shock, inflammation, and ischemia/reperfusion injury. *Pharmacological Reviews*.

[49] Hanaka T., Kido T., Noguchi S. (2019). The overexpression of peroxiredoxin-4 affects the progression of idiopathic pulmonary fibrosis. *BMC Pulmonary Medicine*.

