# Supplementary material for: Overexpression of PRDX4 Modulates Tumor Microenvironment and Promotes Urethane-Induced Lung Tumorigenesis
Source: Oxid Med Cell Longev. 2020 Dec 28;2020:8262730. doi: 10.1155/2020/8262730 (PMC7785354; doi:10.1155/2020/8262730)
Supplement: Supplementary Materials — Supplementary Table 1: differential expression transcripts in the whole-transcript array (fold change > 1.5). Supplementary Figure 1: analysis of antioxidant enzyme gene expressions by RT-PCR in tumor tissues; CAT: catalase, SOD: superoxide dismutase. The 2-ΔΔCT method was used for relative quantification of genes. Data are shown in mean ± SD. The independent samples t-test was used for analysis. ∗p < 0.05. [file 8262730.f1.pdf]

Supplementary table 1.

Differential expression transcripts in whole-transcript array (fold change>1.5).

Transcripts Cluster Id	Tg NO.1	Tg NO.2	Tg NO.3	Non-Tg NO.1	Non-Tg NO.2	Non-Tg NO.3	Gene symbol	Fold change (Tg/Non-Tg)
17325938	111.74	120.10	152.88	78.96	78.90	88.18	*Btla*	1.56
17516538	147.19	110.58	169.58	212.43	240.15	197.72	*Ccdc153*	0.66
17475127	172.88	263.17	256.45	135.70	152.52	128.50	*Cd79a*	1.66
17509617	57.87	65.46	45.72	75.22	83.42	108.49	*Cpe*	0.63
17514330	513.06	366.77	439.42	853.46	567.63	776.52	*Gm10719|Gm10721|Gm10718*	0.60
17285846	37.61	32.14	46.95	21.15	26.17	29.30	*Hist1h4d*	1.52
17344126	112.66	124.45	114.29	785.62	210.14	288.32	*Hspa1b|Hspa1a*	**0.27**
17408856	116.84	115.55	85.23	55.50	75.82	74.60	*I830077J02Rik*	1.54
17467486	498.71	295.04	69.47	36.11	43.66	40.24	*Igkv12-44*	**7.19**
17459400	93.41	79.53	69.92	30.65	33.22	46.30	*Igkv3-5*	**2.20**
17467398	84.93	62.47	166.49	37.61	35.71	46.98	*Igkv4-91*	**2.61**
17405478	125.09	111.04	77.56	58.53	70.98	66.47	*P2ry13*	1.60
17424880	70.21	97.40	98.83	53.83	61.69	56.82	*Pax5*	1.55
17514822	54.52	38.03	52.83	82.02	64.49	76.58	*Taf1d*	0.65

Fold change >2.0 was emphasized in bold.

Supplementary figure 1.

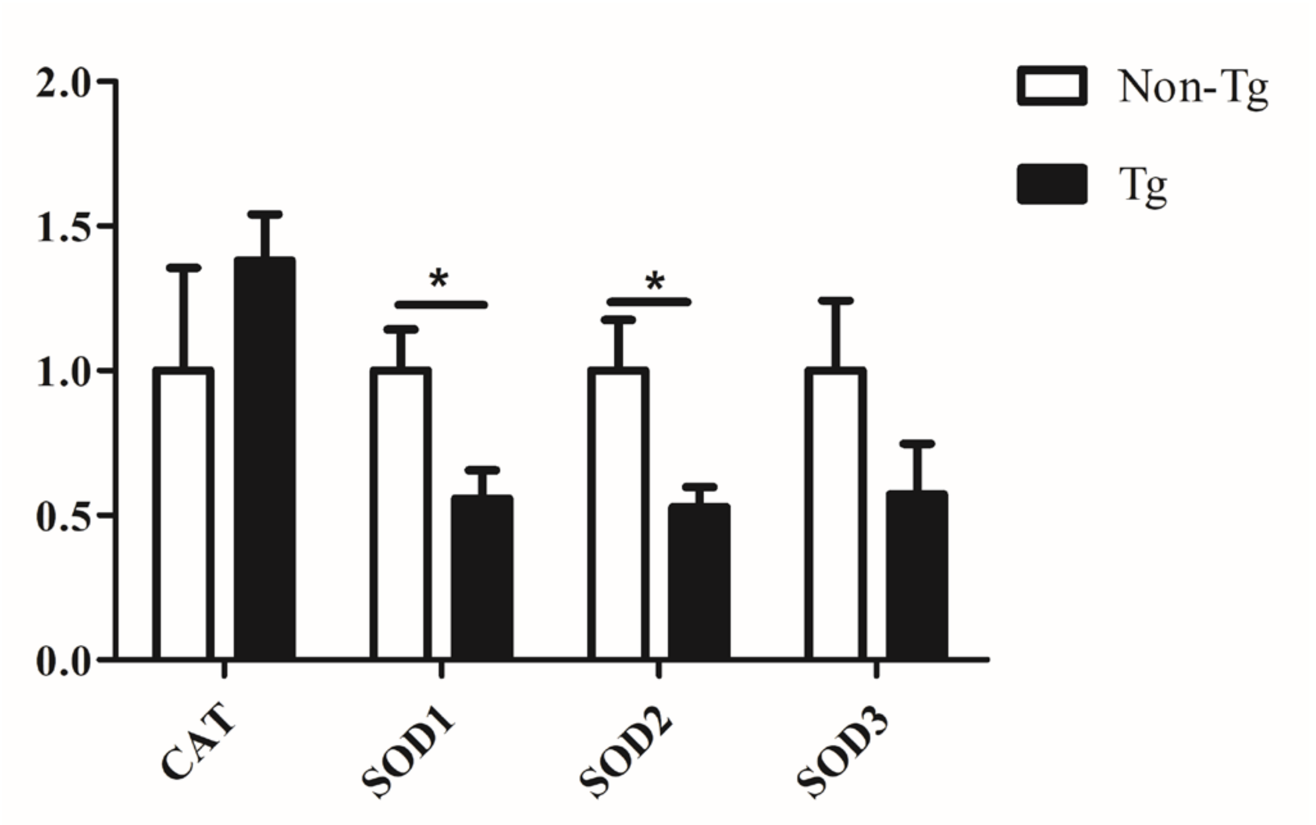

Supplementary figure 1. Analysis of anti-oxidant enzyme gene expressions by RT-PCR in tumor tissues. *CAT*: catalase, *SOD*: superoxide dismutase. $2^{-\Delta\Delta CT}$ method was used for relative quantification of genes. Data are shown in Mean±SD. Independent-Samples t-test was used for analysis.*$p<0.05$.
